# Analysis types and quantification methods applied in UHPLC-MS metabolomics research: a tutorial

**DOI:** 10.1007/s11306-024-02155-6

**Published:** 2024-08-07

**Authors:** Richard D. Beger, Royston Goodacre, Christina M. Jones, Katrice A. Lippa, Oleg A. Mayboroda, Donna O’Neill, Lukas Najdekr, Ioanna Ntai, Ian D. Wilson, Warwick B. Dunn

**Affiliations:** 1 National Center for Toxicological Research, US Food and Drug Administration, Jefferson, AR 72079 USA; 2 Department of Biochemistry, Cell and Systems Biology, Centre for Metabolomics Research, Institute of Systems, Molecular, and Integrative Biology, University of Liverpool, Liverpool, L69 7ZB UK; 3 Office of Advanced Manufacturing, National Institute of Standards and Technology, Gaithersburg, MD 20899 USA; 4 Office of Weights and Measures, National Institute of Standards and Technology, Gaithersburg, MD 20899 USA; 5 Center for Proteomics and Metabolomics, Leiden University Medical Centre, Leiden, The Netherlands; 6 School of Biosciences and Phenome Centre Birmingham, University of Birmingham, Birmingham, UK; 7 Faculty of Medicine and Dentistry, Institute of Molecular and Translational Medicine, Palacký University Olomouc, 779 00 Olomouc, Czech Republic; 8 BioMarin Pharmaceutical Inc., San Rafael, CA USA; 9 Computational and Systems Medicine, Department of Metabolism, Digestion and Reproduction, Imperial College London, London, UK

**Keywords:** Metabolomics, UHPLC-MS, Quantification, Untargeted, Semi-targeted, Targeted, Validation

## Abstract

**Background:**

Different types of analytical methods, with different characteristics, are applied in metabolomics and lipidomics research and include untargeted, targeted and semi-targeted methods. Ultra High Performance Liquid Chromatography-Mass Spectrometry is one of the most frequently applied measurement instruments in metabolomics because of its ability to detect a large number of water-soluble and lipid metabolites over a wide range of concentrations in short analysis times. Methods applied for the detection and quantification of metabolites differ and can either report a (normalised) peak area or an absolute concentration.

**Aim of review:**

In this tutorial we aim to (1) define similarities and differences between different analytical approaches applied in metabolomics and (2) define how amounts or absolute concentrations of endogenous metabolites can be determined together with the advantages and limitations of each approach in relation to the accuracy and precision when concentrations are reported.

**Key scientific concepts of review:**

The pre-analysis knowledge of metabolites to be targeted, the requirement for (normalised) peak responses or absolute concentrations to be reported and the number of metabolites to be reported define whether an untargeted, targeted or semi-targeted method is applied. Fully untargeted methods can only provide (normalised) peak responses and fold changes which can be reported even when the structural identity of the metabolite is not known. Targeted methods, where the analytes are known prior to the analysis, can also report fold changes. Semi-targeted methods apply a mix of characteristics of both untargeted and targeted assays. For the reporting of absolute concentrations of metabolites, the analytes are not only predefined but optimized analytical methods should be developed and validated for each analyte so that the accuracy and precision of concentration data collected for biological samples can be reported as fit for purpose and be reviewed by the scientific community.

## Introduction

The development and application of metabolomics and lipidomics in biological research has significantly progressed in the last twenty years (Han & Gross, 2022; Kell & Oliver, 2016). Developments in new scientific instruments (Hu et al., 2005; Michopoulos et al., 2009), software and bioinformatic tools (Misra, 2021) and cloud computing (Mendez et al., 2019) have allowed metabolomics and lipidomics to be applied across many areas including the biotechnology, agricultural and clinical sciences arenas (Hall, 2018; Kennedy et al., 2018; Yang et al., 2019).

Of all of the analytical approaches applied in metabolomics and lipidomics research, ultra high performance liquid chromatography coupled to mass spectrometry (UHPLC-MS) is one of the most frequently used (for examples see Cajka & Fiehn, 2014; Perez de Souza et al., 2021; Dunn et al., 2011; Plumb et al., 2023; Want et al., 2010; Zheng et al., 2020; Züllig et al., 2020). UHPLC-MS currently provides the potential for rapid and relatively comprehensive analysis of complex biological samples based on various stationary phase chemistries that allow the retention and separation of polar, mid polar and non-polar metabolites/lipids combined with their sensitive detection. These instrumental capabilities have enabled a range of different UHPLC-MS analysis strategies, with different experimental objectives and types of metabolite quantification to be developed and widely applied in metabolomics and lipidomics research.

In this tutorial we aim to (1) define similarities and differences between different analytical approaches applied in metabolomics and lipidomics and (2) define how the abundance of endogenous and exogenous metabolites can be reported as a (normalised) peak area or concentration and report the advantages and limitations of approaches reporting concentration data.

## Different types of analyses are applied in metabolomics research

Three generalised types of assays are applied in metabolomics research as discussed below and which are summarised in Table 1. The two commonly applied types of assays are untargeted and targeted assays. Untargeted assays are discovery-based studies where no target metabolites are listed prior to data collection. In such analyses, hundreds or thousands of mass/retention time pairs (features) corresponding to metabolites are detected but only relative amounts based on (normalised) peak areas, and not absolute concentrations (e.g. micromoles.L^−1^), are reported. At the other end of the assay type spectrum, targeted assays report data only for a small number (usually tens not hundreds) of preselected metabolites defined prior to data collection and absolute concentrations (not normalised peak areas) are reported for all of these metabolites. Targeted assays are analytically validated to a much higher level compared to untargeted assays in relation to properties such as linear calibration ranges (with external and internal standardisation), limits of detection (LOD) and upper and lower limits of quantification (ULOQ & LOQ), linear range, recovery, precision, accuracy and stability. Validated assays also usually employ quality control samples of known concentration of the target analytes. The third type of assay, less frequently applied, is defined as a semi-targeted assay. This assay type applies some characteristics of both untargeted and targeted assays. For example, these assays can consist of the analysis of many tens to hundreds of metabolites (a criteria for untargeted assays) though the list of targeted metabolites is pre-defined (a criteria for targeted assays). A second example relates to whether the abundance of a metabolite is reported as a (normalised) peak area (a criteria for untargeted assays) or as a concentration (a criteria for targeted assays) or a mixture of both (for example, SQUAD approaches (Amer et al., 2023) where some metabolites are reported as concentrations and some are reported as normalised peak areas).

**Table 1:** Characteristics of untargeted, semi-targeted and targeted analyses applied in metabolomics research

Analysis characteristic	Untargeted	Semi-targeted	Targeted
Number of metabolites typically detected	Hundreds or low thousands of metabolites detected dependent on sample type, sample extraction method and UHPLC assay applied	Tens or hundreds of metabolites are detected	One to tens of metabolites
Level of quantification	1. (Normalised) chromatographic peak area reported. 2. No metabolite concentrations reported. 3. Chromatographic peak area applied as a surrogate of concentration	1. Absolute concentration of some metabolites may be reported though typically most metabolites are reported as peak areas. 2. Use of isotopically-labelled internal standards and construction of calibration curves possible. 3. Typically, multiple metabolites are quantified for each single calibration curve	1. Absolute concentration of metabolites reported. 2. Use of isotopically-labelled internal standards and construction of calibration curves with authentic chemical standards. 3. Typically, one calibration curve is constructed for each metabolite
Metabolite identification	1. Some metabolites expected to be detected are known. 2. The chemical structures of many other metabolites are not known prior to the assay. 3. Data acquired during the assay are applied to annotate/identify metabolites	1. Most/all metabolites to be detected and reported are typically known before data is collected. 2. RT, MS1 and MS/MS data are applied to confirm metabolite identity. 3. The chemical structures of some other metabolites are not known prior to the assay and data acquired during the assay are applied to annotate/identify metabolites	1. Metabolites to be detected are known before data is collected. 2. RT, MS1 and MS/MS data are applied to confirm metabolite identity
Time to biological interpretation	Requires metabolite annotation/identification and typically some but not all metabolites are annotated/identified. Can be a time-consuming process	Metabolites are known prior to data collection with processes for biological interpretation typically available which are time-efficient	Metabolites are known prior to data collection with processes for biological interpretation typically available which are time-efficient
Biological bias to metabolites detected	1. Smallest level of bias introduced when multiple complementary UPLC assays are applied. 2. Any bias is related to the concentration of metabolites, metabolites present at low concentration (sub-nanomolar) are not routinely detected	Bias is introduced as metabolites are chosen for assay based on availability of chemical standards and not necessarily to expected metabolic changes in study	Bias is introduced as small number of metabolites are chosen for assay. 2. However, strong evidence from previous studies ensure biologically important metabolites are detected
Type of assay	Discovery/hypothesis generating	Hypothesis generating/hypothesis testing	Hypothesis testing/translation

Further information on each strategy is included below.

### Untargeted assays

- This type of assay starts from a position where the metabolites (targets) of biological interest are not known and instead the objective of this study is to collect reproducible data on a wide range of chemical constituents (endogenous metabolites, exogenous metabolites, exposome, chemical contaminants) without knowing the chemical structures of the chemicals to be reported before data is collected.
- Sample preparation is typically applied to ensure recovery of a wide range of metabolites and chemicals with no attempt to select or isolate target metabolites (using solid phase extraction for example) as can be applied in a targeted assay.
- Untargeted assays are used as part of hypothesis generation by screening of a large number of metabolites to highlight (and annotate, or where possible identify) the smaller number of metabolites that appear to be biologically important in relation to the study question (which is typically to identify a biomarker or understand molecular mechanisms). Typically, hundreds or thousands of features (*m/z*-retention time pairs) are detected in the samples. Importantly, these approaches are sometimes referred to as global strategies though no single analytical instrument or assay can detect all chemical constituents. Thus, it is inevitable that some biases are introduced in sample preparation and data acquisition methods (for example, highly volatile compounds are not detected by UHPLC-MS but can be detected by GC–MS).
- As the constituents are often not known prior to data acquisition, and in many cases only tentatively structurally annotated, the construction of calibration curves are not performed and all that can reasonably be done in this instance is to report the intensity of a chemical’s signal as a chromatographic peak area. Any isotopically-labelled internal standards spiked into the biological samples, extraction solvents or extraction solutions are not used for quantification but are instead used to demonstrate the analytical method repeatability by e.g., monitoring retention time stability, injection volumes etc. (Broadhurst et al., 2018; Lewis et al., 2016).
- Validation of untargeted assays is limited to repeatability, stability (sample and instrumental) and, to some extent, metabolome coverage (Zelena et al., 2009). Repeatability relies on the assay, solvents, chromatographic separation and mass spectrometer performance.
- Metabolite annotations and identifications are derived after data acquisition from the data collected. The reporting of metabolite annotations or identifications and the level of confidence can vary depending on the types of data used and how data are compared to libraries of retention times (RT or t_R_), MS/MS mass spectra and databases of known or predicted metabolites. Confidence ranges from a feature being reported as an unknown metabolite to high confidence identification based on comparison of RT and MS/MS data to a pure chemical standard analysed applying the same assay as was applied for the biological sample. Four levels of reporting confidence have been recommended by the Metabolomics Standards Initiative [Sumner et al., 2007] and more recently a five-level system for LC–MS only was published [Schymanski et al., 2014].

### Targeted assays

- This type of analysis starts from a position where the metabolites of biological interest (targets) are specified prior to the study’s data collection and the number of metabolites targeted are typically small in number, from one to a few tens of metabolites.
- As the metabolites are known, they can be purchased (or synthesised) and applied to develop/validate an assay and to construct a calibration curve for each metabolite. If the metabolite(s) are not available then a validated targeted assay cannot be developed and applied (using a surrogate metabolite for reporting concentrations of high accuracy is not recommended by the authors).
- The chemical structure of the metabolites are known before samples are analysed and therefore confirmation of the structure is not required. Monitoring of RT and metabolite-specific product ions and comparison to the data collected for a chemical standard are applied for confirmation of the identity of the analyte.
- Sample preparation for targeted assays can be more intensive so as to prepare a solution for analysis which contains the analytes but for which many, or all, of the other matrix components have been removed. There are a range of sample preparation techniques available including liquid–liquid extraction, solid phase extraction and Quick Easy Cheap Effective Rugged, Safe (QuEChERS) (for example, see Kanu, 2021 and Keevil, 2016). Chemical derivatisation can also be applied in targeted assays to increase specificity and selectivity (for example, see Huan et al., 2018 and Ubhi et al., 2013).
- The use of isotopically labelled internal standards are typically applied with the goal to compensate for variability introduced during sample preparation and data collection. The chemical standard and internal standard are applied to report a high accuracy concentration for each metabolite, which we will define as absolute quantification in this manuscript.
- Where isotopically labelled internal standards are not available the use of external calibration curves may be the only alternative for quantification in targeted assays.
- Traditionally, targeted assays are performed on triple quadrupole mass spectrometers using multiple reaction monitoring (MRM) and through monitoring of product (fragment) ions for performing quantification and confirmation of metabolite identity (McMillen et al., 2023). More recently, the use of HRAM instruments are being applied to perform quantification, where either precursor or product ions are applied for quantification (Hines et al., 2017). When using e.g., MRM, it is important to ensure that no other co-eluting compounds have one or multiple similar precursor or product ions during method development as this can result in in reporting a higher metabolite concentration than is present in the biological sample (Jia et al., 2023). To ensure the targeted assay is fit for purpose, it should be experimentally validated to a level dependent on how and where the data will be applied (e.g. academic laboratory vs. clinical biochemistry laboratory). See Sect. 4 below for information on validation approaches.
- This type of analysis is applied in the testing of biological hypotheses (Li et al., 2018) or in translational aspects of research, for example, where research is moved from the research laboratory in to the clinical biochemistry laboratory (Keevil, 2016). Targeted LC–MS/MS assays are now routinely applied across different scientific disciplines including clinical diagnostics (Seger & Salzmann, 2020), pharmaceutical research (Beccaria & Cabooter, 2020), water quality monitoring (Milić et al., 2018; Nakhjavan et al., 2021) and food safety (Steiner et al., 2021).

### Semi-targeted assays

- This type of assay can be applied to fulfil a variety of objectives and has resulted in a number of different definitions including hybrid analysis (Chen et al., 2020), wide-targeted metabolomics (Han et al., 2024) and pseudotargeted metabolomics (Zheng et al., 2020).
- The assay type can have the characteristics of both untargeted and targeted assays. For example, tens to hundreds of metabolites are reported (a criteria for untargeted assays) though some or all metabolites can be reported with concentrations (a criteria for targeted assays). There are different objectives for semi-targeted assays and there are basically three different approaches (types one to three) which are defined in Table 2 and below.
- Sample preparation approaches applied in untargeted assays are often used. Isotopically labelled internal standard(s) can be spiked into samples, extraction solvents or post-extraction solutions to aid in the quantification of metabolites (as would be applied for a targeted assay) and/or to be used for assessing the reproducibility of sample preparation and data acquisition in a quality control process (as would be applied in an untargeted assay) (Broadhurst et al., 2018; Lewis et al., 2016). The analytical validation applied (if any) can also be less rigorous than that performed in targeted assays.
- Assay type one is performed using a defined list of metabolites typically constructed with hundreds or low thousands of metabolites. For this RT and/or MS1 and/or MS/MS data are collected using chemical standards and are applied to *confirm* the metabolite identity as is applied in a targeted assay rather than *structurally determine* the metabolite identity as performed in untargeted studies. Importantly, only those metabolites on the defined list are reported and so only confirmation of structure for these metabolites is required. These methods are reported as semi-targeted because they have a defined and large target list but report signal intensities and not concentrations of metabolites (see Che et al., 2018 for an example). Of note, the commercial metabolomics service provider Metabolon applies this approach though the full methods applied are not available publicly and so cannot be verified (see (Metabolon, 2022)).
- Assay type two also focuses on hundreds of metabolites but here an absolute concentration will be reported instead of a (normalised) peak area as for assay type one. These assays are based on RT and/or MS1 and/or MS/MS data obtained using chemical standards which are used to *confirm* metabolite identity as is applied in a targeted assay. Absolute quantification of *all* of these metabolites is performed by applying single or multiple point calibration curves to report concentrations; typically a single point calibration is applied to calculate the concentration of many of the metabolites (Biocrates, 2022; Mahmoudian-Dehkordi et al., 2019; Thompson et al., 2019). The strengths and weaknesses of single and multiple point calibration curves are discussed in Sect. 3.3.
- Assay type 3 also involves a specific list of target metabolites to be determined, perhaps a metabolic pathway or a class of metabolites. Here RT and/or MS1 and/or MS/MS data are collected using chemical standards to *confirm* the metabolite identity as is applied in a targeted assay. Absolute quantification of *a subset but not all metabolites* is performed applying single or multiple point calibration curves. For all other metabolites, the component signal is reported as for untargeted assays or component signals are normalised to one of the internal standards applied for absolute quantification to report a normalised response. This type of analysis can be applied to target a smaller number of metabolites of known biological interest (for hypothesis testing) as applied for targeted assays while simultaneously collecting data for other unknown targets (hypothesis generation) as is applied in untargeted assays. This assay is not frequently applied currently but is an assay type expected to be observed more frequently in the future (Amer et al., 2023).

**Table 2:** Characteristics of different types of semi-targeted analyses applied in metabolomics research

Analysis characteristic	Type 1	Type 2	Type 3
Number of metabolites typically detected	Hundreds or low thousands of metabolites. Either all metabolites reported are targeted or of metabolites reported some were targeted and some were not targeted	Hundreds or low thousands of metabolites. All metabolites are targeted	Hundreds or low thousands of metabolites. All metabolites are targeted
Level of quantification	(Normalised) chromatographic peak area reported (no metabolite concentrations reported)	Absolute quantification to report mass concentrations with use of single point or multiple-point calibration curves. Typically one calibration curve is applied to quantify multiple metabolites	1. Absolute quantification to report mass concentrations for a subset of metabolites (typically less than 100) with use of single point or multiple-point calibration curves. Typically one calibration curve is applied to quantify multiple metabolites. 2. (Normalised) chromatographic peak area reported for other targeted metabolites (no metabolite concentrations reported)
Metabolite annotation/identification or confirmation of metabolite identity	RT and/or MS1 and/or MS/MS data are collected using chemical standards and are applied to *confirm* the metabolite identity	RT and/or MS1 and/or MS/MS data are collected using chemical standards and are applied to *confirm* the metabolite identity	RT and/or MS1 and/or MS/MS data are collected using chemical standards and are applied to *confirm* the metabolite identity for targeted metabolites and structural annotation for untargeted metabolites
Time to biological interpretation	Metabolites are known prior to data collection with processes for biological interpretation typically available which are time-efficient	Metabolites are known prior to data collection with processes for biological interpretation typically available which are time-efficient	Metabolites are known prior to data collection with processes for biological interpretation typically available which are time-efficient

## Types of quantification

Definitions of processes applied in quantification are defined in Table 3. In metabolomics studies we are measuring the amounts of metabolites, relative quantification in untargeted and some semi-targeted assays or absolute quantification in targeted and some semi-targeted assays. Both types of data can be applied to perform a comparison between two or more biological groups (for example, a healthy human population is compared to a human population with a specific disease). There are different approaches applied to represent the amount of a metabolite. Some approaches report a concentration (absolute quantification) by comparing the sample response to a calibration curve constructed with authentic chemical standards, these approaches differ by the confidence in the accuracy and precision of the reported concentration. Other approaches report a surrogate to concentration (relative quantification) which is typically a single ion chromatogram peak area (which is not compared to a calibration curve constructed with chemical standards to report a concentration). A number of review articles have discussed the principles, advantages and limitations of different quantification strategies (Cajka & Fiehn, 2016; Lu et al., 2017; Rampler et al., 2021).

**Table 3:** List of definitions applied in endogenous metabolite quantification

Term or item	Definition
Bioanalytical method development (FDA, 2018)	The purpose of bioanalytical method development is to define the design, operating conditions, limitations, and suitability of the method for its intended purpose and to ensure that the method is optimized for validation
Bioanalytical method validation (FDA, 2018)	Bioanalytical method validation proves that the optimized method is suited to the analysis of the study samples
Quantification (Shor, 2008)	The act of giving a numerical value to a measurement of something, that is, to count the quanta of whatever one is measuring. Quantification produces a standardized form of measurement that allows statistical procedures and mathematical calculations
Normalisation (Di Guida, 2016)	Normalisation is applied to correct for unwanted peak intensity differences derived from variation introduced during sample collection, preparation and analysis and to stabilise the variance within the dataset
Mass concentration	Ratio of the mass of a solute present in the solution to the volume of the solution
Molar concentration	Ratio of the number of moles of a solute present in the solution to the volume of the solution
Reference material (ISO, 2015)	Material, sufficiently homogeneous and stable with respect to one or more specified properties, which has been established to be fit for its intended use in a measurement process
Calibration (Gonzalez, 2020)	Calibration determines the relationship between the analytical response from an instrument and the analyte concentration. This relationship allows then to determine the concentration of the analyte in an unknown sample. There are several methods of calibration
External calibration	For many analytical methods it is possible to run a series of standards, construct a calibration curve and then determine unknown samples from that curve. This is called external calibration
Internal standard	An internal standard (I.S.) in analytical chemistry is a substance that is similar to the analyte that is added in a constant amount to the blank, the standards, and the samples. Internal standards are useful to compensate for changes in extraction efficiency, detector response due to sample loss during other sample preparation steps, fluctuations in sample analyzed, or changes in detector response due to different flow rates
Standard addition	The standard addition method is similar to the external calibration method in that the concentration of an analyte is determined by comparison to a set of standard solutions of the analyte. However, in the standard addition method, the standard is added to the sample to correct for ‘matrix effects’ (a change in the analytical signal caused by anything in the sample other than the analyte). This is called “spiking.”

There are three important components of each type of assay described in Sect. 2; (a) annotation/identification of the metabolite(s) from raw data (untargeted and some semi-targeted assays) or confirmation of their identification (targeted and some semi-targeted assays), (b) quantification and reporting of concentrations (targeted and some semi-targeted assays) or (normalised) peak areas (untargeted and some semi-targeted assays) and (c) the level of confidence in both (a) and (b).

For reporting of absolute concentrations, the accuracy of the reported concentration in relation to the actual concentration is highly important to allow the user of the data to apply the data appropriately. For most semi-targeted and targeted assays the accuracy is not routinely reported for these data, **but** the scientific community assumes that any targeted assay will report a concentration with high accuracy. The accuracy of an assay where metabolite concentrations are reported is highly dependent on factors including the analyst, assay, method of concentration calibration (e.g. single point versus multiple point calibration) and level of assay validation. For example, the accuracy of reported concentrations for the amino acid tryptophan when using a calibration curve constructed with the amino acid leucine should be assumed to be of low accuracy unless experimentally demonstrated otherwise during assay validation (see Fig. 2 for a visual example of this). This point highlights the need for consideration of which external standards and internal standards to be applied for quantification and reporting of concentrations. A discussion on considerations for the choice of internal standards is included in Sect. 3.7 below. For external standards it is recommended to use the structurally identical analyte because a change in chemical structure (even by the addition or removal of a methyl group or a different position of a hydroxyl group) can change retention time and ionisation efficiency and therefore quantification accuracy. The different processes and types of quantification are discussed below.

### Chromatographic peak areas and normalisation

Peak areas before or after normalisation are typically applied in untargeted and some semi-targeted analyses to allow relative changes in amount to be reported. Normalisation is a calculation performed to determine the contribution of the response for one metabolite in relation to either (i) all other metabolites in the dataset or (ii) all other samples in the dataset. Normalisation can be performed before data collection (e.g. normalisation to wet tissue weight or urine osmolarity; (Chetwynd et al., 2016; Southam et al., 2020) or after data collection (e.g. PQN) (Di Guida et al., 2016). A range of normalisation methods have been compared for pre-analysis normalisation (e.g. Chetwynd et al., 2016; Khodorova et al., 2024) and post-analysis normalisation (Di Guida et al., 2016). In the process of normalisation, chromatographic peak area data are not compared to a calibration curve constructed with a chemical standard and so no concentration can (or should) be reported. This type of quantification is sometimes defined as relative quantification but do not let the word quantification define that a metabolite concentration is reported. Normalisation or relative quantification is normally applied in untargeted analyses and some semi-targeted assays as described above. Importantly, the response or normalised response can demonstrate drift during an analytical batch or batches which can impact on the quality of data. Internal standards and pooled QC samples can be applied to determine whether drift is present and if so to remove the drift observed in these data (Broadhurst et al., 2018).

### Reporting of a concentration by comparison to a reference material

Chromatographic peak area data can be converted to a concentration by comparison to a reference material with defined metabolite concentrations reported (see ISO, 2015 and Lippa et al., 2022). Here the biological samples and reference material are analysed separately in the same analytical batch. The peak area in the biological sample is compared to the peak area and concentration in the reference material to enable the calculation of the concentration in the biological sample. For example, the concentration of tryptophan in the reference material is 200 micromoles.L^−1^ and the measured peak area in the reference material was 200,000. If the peak area in the biological sample was 100,000 or 400,000 then the estimated concentrations would be 100 or 400 micromoles.L^−1^, respectively. This workflow has an unvalidated assumption that the correlation between response and concentration is linear over the range of responses measured for the biological samples, that there are no confounding matrix dependent effects, and that the detector is not saturated at higher concentrations. None of these can be confidently assumed without conducting the appropriate assay validation and reporting the validation outcomes and therefore high accuracy in reported concentrations should not be assumed. Yu and Huan have demonstrated compression or inflation of signal ratios even in the linear response range and demonstrated that even diluting samples in to the linear response range is not always appropriate. They proposed a metabolic ratio correction (MRC) strategy to minimize signal ratio bias in untargeted metabolomics for accurate relative quantitative comparison based on dilution analysis of a pooled QC sample (Yu & Huan, 2021).

### External standard calibration

This method operates through the construction of a calibration curve(s) with an authentic chemical standard(s). The chromatographic-MS (MS1) or chromatographic-MS/MS (MS2, product ion) peak area data for the chemical standards present at different concentrations in different calibration solutions are used to construct a calibration curve (concentration *vs.* peak area). The chromatographic-MS or MS/MS peak areas for the metabolite in the biological samples are then compared to the peak areas observed for the calibration curve to calculate a concentration in the biological sample. In targeted assays based on MRM analyses two or more MRM transitions are applied wherever possible, one for the purpose of quantification and one or more for confirmation of the metabolite’s chemical structure (through comparison of product ion ratios for the chemical standard and the metabolite in the biological sample). Calibration of instrument response can be performed with a single point calibration or multiple point calibration. Single point calibration is applied in metabolomics research because it is experimentally easier to apply in relation to preparation of the calibration solution and total analysis times are shorter because multiple calibration solutions are not applied. However, the concentration reported is not necessarily as accurate for a single point calibration as one obtained using a multiple point calibration method. For users of these data this should be clearly reported to avoid confusion; for example, a clinician using these data would expect accurate concentrations to be reported as this is the normal practice for his working environment. The authors do not recommend single point calibration to report accurate concentrations of metabolites. The authors also recommend that where feasible the analyst should use the same analyte as is being quantified for calibration curve construction. However, in some cases (e.g., especially lipidomics) this is not feasible, especially for large numbers of metabolites to be assayed, and so single point calibration has been developed for these applications. In these cases, the assay and reporting of concentrations could be fit-for-purpose (for example, they would be appropriate for a biological discovery study but not for a regulated analytical chemistry laboratory). However, when used a full reporting of methods should be included so as to allow the research community to understand the methods and the influence they may have on the accuracy of the reported concentrations.

A higher accuracy will generally be observed for multiple point calibration and where the responses for the biological samples are observed in the linear range of responses for the calibration curve. The reporting of concentrations which fall in to the linear calibration range is preferred because a higher accuracy will be observed. The reporting of concentrations which fall in to non-linear areas of the calibration curve can be applied with the understanding that accuracy could be lower and that this approach should not be used if the response is saturated (i.e. the response does not change as concentration changes). The authors recommend that where a concentration is reported as higher than the upper limit of the linear calibration range then the sample solution is diluted so that its concentration falls in to the linear range and a dilution factor is applied to correct for the dilution on the metabolite concentration.

External calibration curves can be problematic to prepare as it is generally not possible to find matrices where the endogenous metabolite being determined is absent. This often means that these curves are prepared in either water (or a suitable solvent) or in a surrogate matrix (see Sect. 3.8). As neither of these alternatives is likely to accurately compensate for matrix effects in the samples under study, techniques such as “overspiking” or “standard addition” with an authentic standard at known concentration(s) can be applied to confirm the accuracy of the derived concentrations (these procedures are described in Sect. 3.5 below).

### The use of isotopically-labelled internal standards

This method can operate in a similar way to external standard calibration but internal standards, at the same concentration, are spiked in to each calibrant solution and biological sample prior to analysis. The response ratio is used as the response parameter applied for the calibration curve, instead of the chromatographic peak area. The response ratio is calculated as analyte peak area/internal standard peak area. Any analytical error introduced that influences the peak area and therefore concentration reported for the metabolite should equally be observed for an appropriately chosen internal standard, i.e., the effect on the metabolite peak area will be replicated for the internal standard peak area. This is dependent on at which stage the internal standard is spiked in the extraction process; for example, if an internal standard is added after extraction then it will not compensate for errors introduced during the sample extraction process itself. Internal standards can be added at different stages of sample preparation, typically they are either introduced in to the biological sample as the first step of the sample preparation process, included in the extraction solution and so introduced during the extraction process or are added to the sample extract solution after extraction as the final step before data collection. Different internal standards can be added at one or each of these three steps also; for example, one internal standard is added to the biological sample before extraction and a different internal standard is added to the extraction solution after extraction has been completed to determine variation and errors at different stages of the analytical process. The concentrations of the internal standards should fall within the linear calibration range of the assay for each metabolite being measured. The choice of internal standard will be discussed in Sect. 3.7 below.

The construction of a calibration curve using serial dilution of isotopically-labelled internal standards and not non-isotopically labelled metabolites can also be applied to report concentration data. This quantification method is referred to as isotope dilution mass spectrometry (IDMS) (Ryan et al., 2023). Pure isotopically-labelled internal standard(s) can be purchased separately and prepared as a multi-component solution or alternatively ^13^C-labelled cell extracts can be used (Hermann et al., 2018; Jaber et al., 2023; Gleichenhagen et al., 2013).

### Standard addition

Briefly, multiple aliquots of the same biological sample have different concentrations of the target metabolite(s) spiked in to them (typically 4–8 different concentrations) followed by analysis of the unspiked and spiked samples. Once the data are plotted (concentration on x-axis *vs.* response on y-axis), the correlation is assessed for linearity and, if linear, the intercept on the x-axis is defined as the metabolite concentration in the biological sample. This type of quantification requires analysis of the same sample (unspiked and overspiked) multiple times, therefore increases the analysis time for each sample and is infrequently applied in metabolomics studies. It should be noted that standard addition methods will not provide a calibration curve across the entire linear calibration range but only for concentrations from the sample concentration to the upper limit of the linear calibration range.

### Accuracy of concentrations reported

**Fig. 1:**
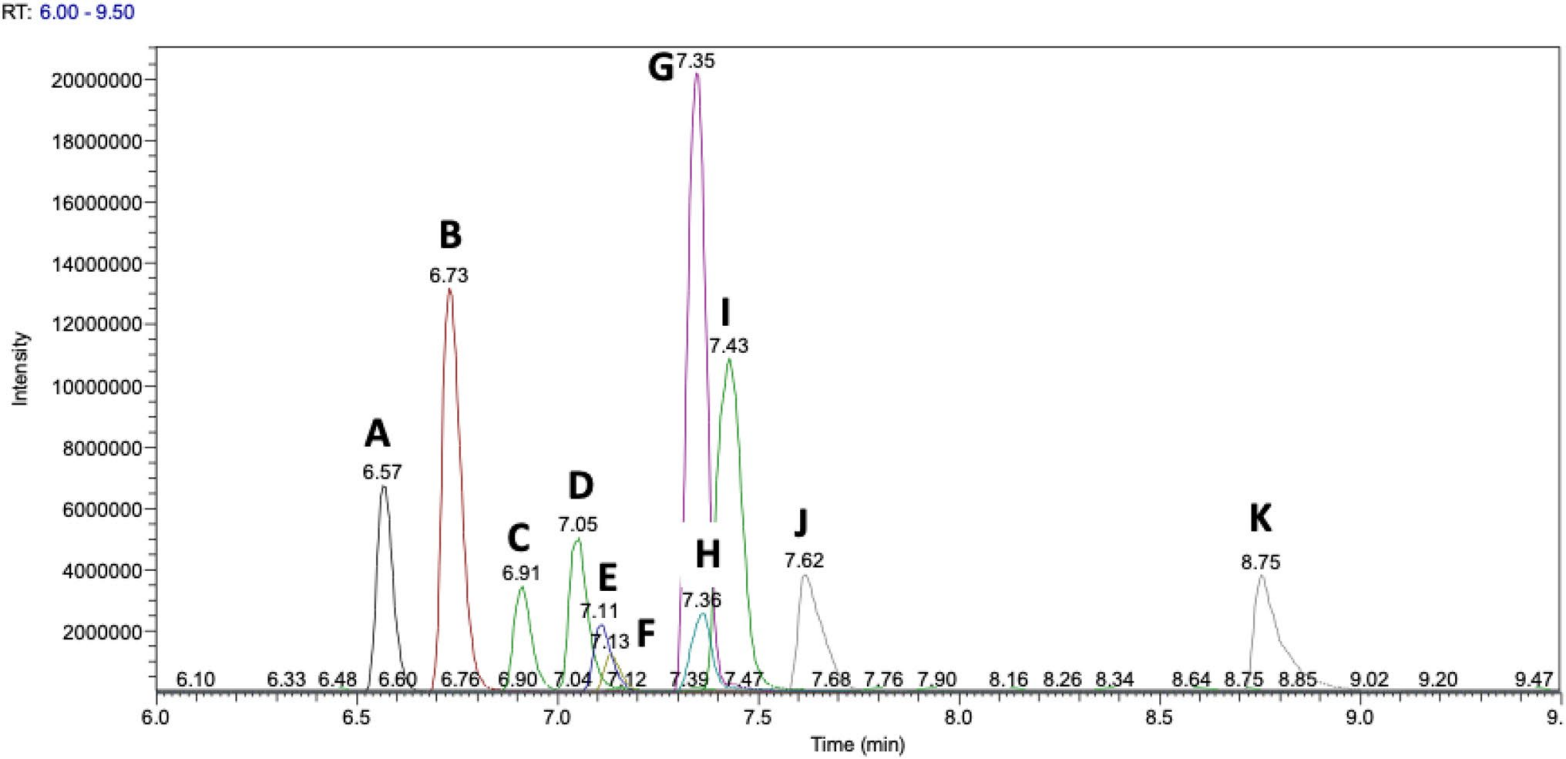
Single ion mass chromatograms for eleven amino acids present at a concentration of 10 µg.mL^−1^ and analysed applying the same UHPLC-MS assay in a single injection analysis showing the difference in intensity for compounds of the same metabolite class, but different chromatographic and ionization properties. The amino acids are (A) lysine (6.57 min), (B) trans-4-hydroxyproline (6.73 min), (C) valine (6.91 min), (D) tryptophan (7.05 min), (E) leucine (7.11 min), (F) tyrosine (7.13 min), (G) proline (7.35 min), (H) methionine (7.36 min), (I) phenylalanine (7.43 min), (J) isoleucine (7.62 min) and (K) glutamine (8.75 min)

**Fig. 2:**
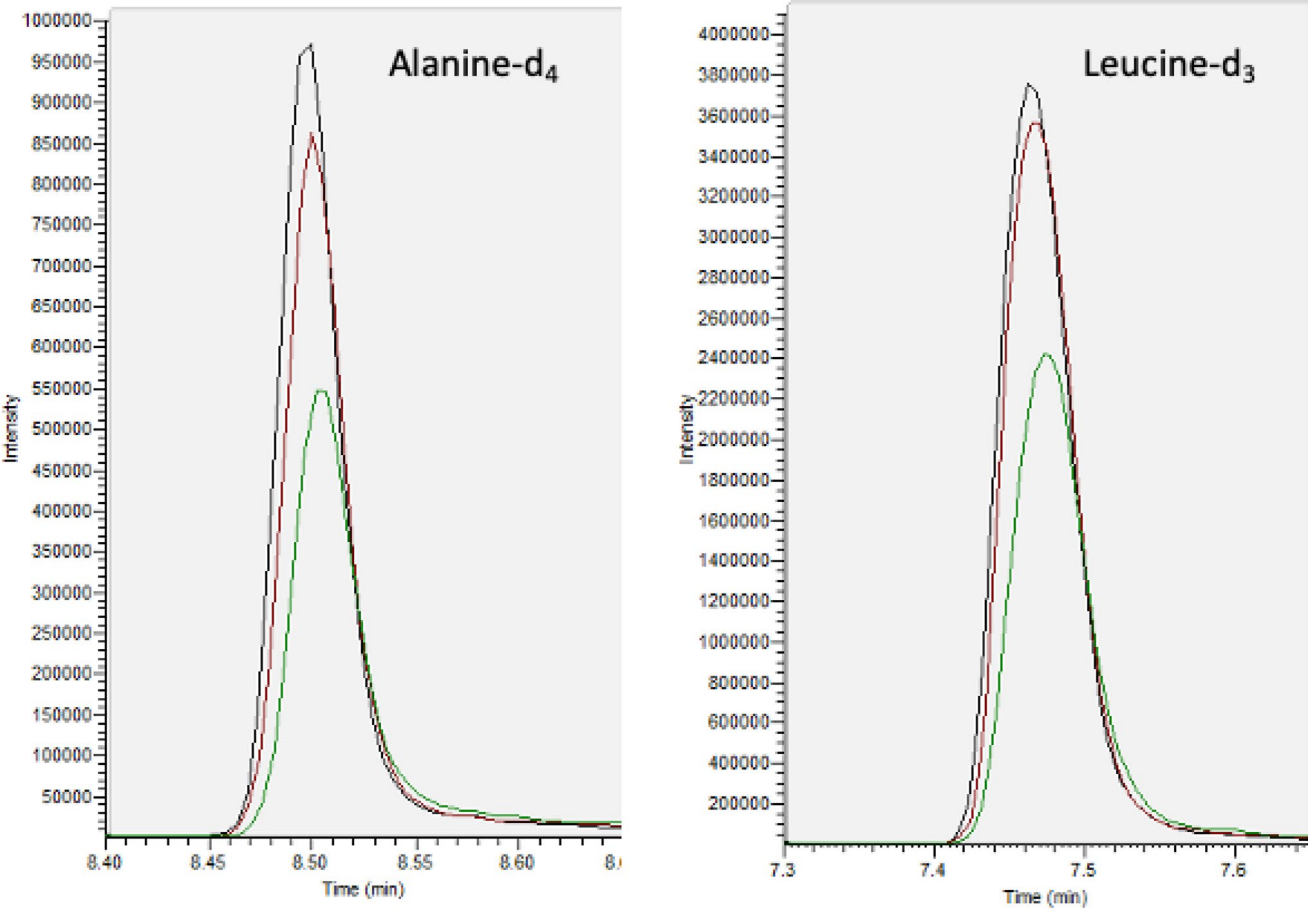
Single ion mass chromatograms for two internal standards spiked in to water (black), human plasma (red) and human urine (green) to the same final concentration and analysed applying the same UHPLC-MS assay in a single injection analysis demonstrating the effects of sample matrix-derived ion suppression on peak intensity

In the sections above we have discussed experimental aspects which contribute to the accuracy of a reported concentration, we will continue these discussions here. At the start of this section we described that high accuracy in reporting of concentrations will not be observed if a calibration curve constructed for one metabolite is applied to report a concentration for another metabolite. However, this strategy is applied in metabolomics and lipidomics and therefore data for these assays can not be viewed as highly accurate but are normally highly reproducible. The Sciex Lipidyzer uses 50 spiked internal standards to quantify up to 1100 lipids in 13 lipid classes (Sciex, 2022) and other similar approaches have been reported by the lipidomics community (Huynh et al., 2019). The Biocrates kits apply a single calibration curve for absolute quantification of multiple metabolites (Biocrates, 2022). When these approaches are validated to demonstrate that they are fit-for-purpose for the study objectives and are applied appropriately they can provide reproducible concentrations across batches of data collected within a laboratory over months or years or for data collected across different laboratories (for example, see (Thompson et al., 2019)). However, just because a concentration is reported it does not mean that the concentration reported is an accurate measurement of the metabolite’s concentration in the biological sample. For example, the actual concentration of metabolite X is 300 µmoles.L^−1^ but using a calibration curve for metabolite Y it is reported as a concentration of 150 µmoles.L^−1^. Typically, the calibration curve for one metabolite is different to the calibration curve of another metabolite in relation to linear calibration range, slope of curve as well as the limit of detection/limit of quantification (LOD/LOQ). As a demonstration, prepare a solution containing the same concentration of twenty amino acids and analyse this solution either by direct infusion into a mass spectrometer or by UHPLC-MS. The peak areas reported for each metabolite will normally be different and so one calibration curve can not be used to report an accurate concentration for all metabolites. See Fig. 1 for a single ion chromatogram of eleven amino acids present at the same concentration which demonstrates different peak heights/areas applying the same assay in the same laboratory). The differences are a result of different ionisation efficiencies of each amino acid and/or for UHPLC-MS analyses ionisation suppression caused by the sample matrix being different across a chromatographic run. For example, in Fig. 1 greater ionisation suppression may be observed in the retention time range 6.8–7.4 min resulting in lower reported peak areas compared to metabolites eluted before 6.8 min or after 7.4 min. The calibration curve for one metabolite in two different sample matrices can also be different in calibration parameters and to test this perform the previous demonstration but for metabolites in three different matrices; pure water, urine and plasma. The mass chromatogram shown in Fig. 2 demonstrates that the same concentration of an internal standard spiked in to three different sample matrices can result in different peak areas being reported.

### Requirements for use of an internal standard

Internal standards are important components of analyses where absolute concentrations are to be reported. The internal standard acts to correct for the introduction of errors or biases during sample preparation and data collection (depending on where in the analytical process the internal standard is spiked as discussed in Sect. 3.4). The use of internal standards can allow concentrations to be reported more accurately and precisely. To be able to act to correct for these errors/biases, especially when using MS-based approaches, the physical and chemical properties of the internal standard must be highly similar (and ideally, to all intents and purposes, identical) to those of the metabolite of interest. This will ensure that chromatographic retention times are identical and that the internal standard will compensate for matrix effects (e.g., ionisation suppression/enhancement during) analysis. Small changes to chemical structure can influence the retention time and electrospray efficiency and therefore decrease the accuracy of a reported concentration. For these reasons the authors recommend using the isotopically labelled metabolite where possible. Not all metabolites have an isotopically labelled internal standard available and therefore a structurally similar internal standard should be chosen and its fitness for purpose assessed experimentally (for example, determine whether similar retention times and electrospray efficiency are observed for metabolite and internal standard). To meet these criteria of similarity, an isotopically labelled analogue of the metabolite of interest is normally applied; for example, for ^12^C_6_ glucose to be assayed a ^13^C_6_ glucose internal standard can be used as it will have the same retention time as endogenous glucose but will be detected at a different mass-to-charge ratio by the mass spectrometer. Isotopes typically incorporated to prepare isotopically-labelled internal standards are ^13^C and/or ^15^N and/or ^2^H. ^13^C and ^15^N isotopically labelled standards are normally more stable and if ^2^H standards are applied then consideration of hydrogen–deuterium exchange (^1^H-^2^H exchange) in solution and how this may influence chemical stability should be experimentally tested. Ideally a mass difference of at least two amu is desirable to ensure that the ^13^C isotope (M + 1) peak of the analyte does not interfere with the parent (M) ion of the internal standard. However, whilst internal standards where e.g. all of the carbon atoms in the molecule have been replaced with ^13^C can be used, as this enrichment does not affect the chromatographic properties of the molecule (similarly for replacement of ^14^N with ^15^N) the same is not true of deuterated compounds where retention times and ionisation efficiency can change significantly if the extent of deuteration is high. Thus, as the similarity in chemical, physical and chromatographic properties between target metabolite and internal standard decreases (i.e., they become more different) then the ability for the internal standard to correct for errors and biases decreases. This is because of the interplay between the chemical structures of metabolite and internal standard as well as differences in retention time which can lead to changes in matrix-derived ionisation suppression as well as ionisation efficiency for metabolite and internal standard. Of course, if you are assaying tens or hundreds of metabolites and because of the cost of or lack of availability of isotopically labelled internal standards then it is not always feasible to apply a different and appropriate internal standard for all metabolites. Here, use of an untargeted approach reporting (normalised) peak areas to determine which compounds may be potential markers and then development of a specific targeted assay for determining their absolute concentration in order to validate the hypothesis is appropriate. Alternatively, a single isotopically-labelled internal standard can be used to report a concentration for multiple lipids of the same class as described in Sect. 3.6.

### Solvent-based or sample matrix-based matrices

Calibration solutions can be prepared in a matrix-free or matrix-matched solutions. This choice can influence the accuracy of the reported concentration as matrix effects can lead to ionisation suppression/enhancement in biological matrices that are not observed in e.g., simple aqueous solutions. Therefore the use of a matrix-free solution for calibration curves and QC samples would not compensate for ion suppression/enhancement observed in the biological samples. Choosing a matrix-matched solution is an obvious choice and works well when studying exogenous metabolites like drugs and their biotransformation products where these exogenous metabolites are not present at any concentration in the matrix-matched solution. However, matrices analysed in metabolomic studies will (almost) always contain endogenous metabolites at physiological concentrations. Thus the absence of analyte free matrices provides difficulties in constructing a calibration curve for the relevant concentration ranges of the metabolite which will report an accurate concentration. A surrogate or synthetic matrix can be prepared and for plasma/serum these include a saline/bovine serum albumin solution or a saline-containing solution for urine (for example, Godoy et al., 2020; Klupczynska et al., 2020). However, the use of these surrogates may only provide a false sense of security as ion suppression is generally associated in plasma/serum with phospholipids which are not present in bovine serum albumin. These factors can be assessed during method validation for targeted, and possibly semi-targeted, methods enabling correction factors to be be applied to increase the accuracy of the reported concentrations; however, their adoption for untargeted methods is very problematic. For targeted methods the use of a structurally identical isotopically labelled internal standard can be used to compensate for these effects. When this is still insufficient, the use of extensive sample preparation (for example, solid phase extraction (Sitnikov et al., 2016) or liquid–liquid extraction) can reduce matrix effects by removing the elements of the sample matrix that cause ionisation suppression; for example, the use of lipid removal SPE plates is routinely adopted for the analysis of endogenous metabolites in serum and plasma (Li et al., 2015; Theodoridis et al., 2008). However, it has to be recognized that all of these approaches require careful evaluation and validation where claims for accurate absolute quantification are made.

### Use of different adducts for quantification

Quantification approaches that involve “using more than one adduct for quantification” are difficult to generalize for large-scale analyses like metabolomics where compounds with varying physicochemical properties, and consequently different ionization properties, are analyzed within a single experiment. Moreover, there is no clear consensus in the field on the optimal and practical approach to address this phenomenon. The most pragmatic approach often adopted, without much theoretical reasoning, is the optimization of sample preparation workflows to minimize adduct formation (e.g., using techniques such as extensive desalting). While simple and seemingly feasible, the inherent complexity of biological samples presents numerous practical challenges. Below, we summarize some alternative solutions that undoubtedly outperform the aforementioned approach but come with increased analysis complexity, time, and financial cost:

- Calculation of response factors for each adduct by comparing the signal of the adducts with the signal of an internal standard; the method could allow for the comparison of metabolite concentrations across samples without absolute quantification.
- If the concentration of the target compound is known, response factors can be calculated by dividing the observed peak area by the known concentration of the parent compound.
- Construction of calibration curves for each adduct type (e.g., [M + Na]^+^, [M + K]^+^) using known concentrations of the target compound; this helps in quantifying the parent compound based on the response factor of each adduct.
- Use of internal standards that ideally mimic the behavior of the target compounds; in theory, the internal standards should form the same types of adducts, thereby compensating for variable ionization efficiencies and matrix effects.

## The requirements for validated targeted and semi-targeted methods including recovery, accuracy, precision and linear calibration range

Targeted methods, and those for metabolites reported with concentrations in semi-targeted methods, should be formally validated to demonstrate that they are fit for purpose to fulfil the analysis objectives. The Food and Drug Administration (FDA) have published that the fit-for-purpose (FFP) concept states that the level of validation should be appropriate for the intended purpose of the study (Food and Drug Administration, 2018) and highlights that the assay validation process as applied to targeted assays helps demonstrate whether the method is fit-for-purpose or not. A number of guidelines are available which define which characteristics of the assay are validated for methods quantifying exogenous drugs and their biotransformation products including from the U.S. Food and Drug Administration (Food and Drug Administration, 2018) and the European Medicines Agency (EMA, 2017). However, similar guidelines for the validation of targeted methods in metabolomics for the quantification of endogenous metabolites have not yet been formulated. Whilst the regulatory guidelines provided by the FDA/EMEA provided a framework that can be adapted to guide the validation of metabolomic assays, methods for endogenous metabolites have different considerations and are subject to different constraints. Thus, unlike assays for exogenous materials such as drugs where blank matrices are easy to obtain, for endogenous metabolites such analyte free matrices are generally not available complicating the preparation of calibration curves and subsequent assessment of recovery, accuracy and precision.

The highest level of method validation is required when data will be applied in regulatory or clinical decisions. As examples of the validation criteria assessed at this highest level, the FDA’s Bioanalytical-Method-Validation-Guidance-for-Industry document provides a useful model. The characteristics and acceptance criteria are defined in Table 4. In general terms there are four criteria being assessed (Food and Drug Administration, 2018):

- Does the method measure the intended analyte? For example, does anything interfere with the measurement, and is the method specific or selective for the analyte?
- What is the variability associated with these measurements? For example, what are the accuracy and precision of the method?
- What is the range in measurements that provide reliable data? For example, what is the sensitivity of the method (e.g., what is the lower limit of quantification (LLOQ) of the method, and what is the upper limit of quantification of the method (ULOQ)?)
- How do sample collection, handling, and storage affect the reliability of the data from the bioanalytical method? For example, what steps need to be followed while collecting samples? Do the samples need to be frozen during shipping? What temperatures are required to store the samples, and how long can the samples be stored?

**Table 4:** Recommendations and acceptance criteria for bioanalytical method validation of chromatographic assays as defined by the food and drug administration (Food and Drug Administration, 2018)

Parameter	Validation recommendations for chromatographic assays	Acceptance criteria
Calibration curve	(1) A blank (no analyte, no IS), a zero calibrator (blank plus IS), and at least six, non-zero calibrator levels covering the quantitation range, including LLOQ in every run	(1) Non-zero calibrators should be ± 15% of nominal (theoretical) concentrations, except at LLOQ where the calibrator should be ± 20% of the nominal concentrations in each validation run
	(2) All blanks and calibrators should be in the same matrix as the study samples	(2) 75% and a minimum of six non-zero calibrator levels should meet the above criteria in each validation run
	(3) The concentration–response relationship should be fit with the simplest regression model	
Quality controls (QC)	(1) For A & P Runs: Four QCs, including LLOQ, low (L: defined as three times the LLOQ), mid (M: defined as mid-range), and high (H: defined as high-range) from at least five replicates in at least three runs	(1) Refer to A & P Runs, Other Validation Runs, and Stability Evaluations
	(2) For Other Validation Runs: L, M, and H QCs in duplicates	
Selectvity	(1) Analyze blank samples of the appropriate biological matrix from at least six individual sources	(1) Blank and zero calibrators should be free of interference at the retention times of the analyte(s) and the IS
		(2) Spiked samples should be ± 20% LLOQ
		(3) The IS response in the blank should not exceed 5% of the average IS responses of the calibrators and QCs
Specificity	(1) The method specificity should be assessed for interference by cross-reacting molecules, concomitant medications, bio-transformed species, etc	(1) See Selectivity above
Carryover	(1) The impact of carryover on the accuracy of the study sample concentrations should be assessed	(2) Carryover should not exceed 20% of LLOQ
Sensitivity	(1) The lowest non-zero standard on the calibration curve defines the sensitivity (LLOQ)	(1) The analyte response at theLLOQ should be ≥ five times the analyte response of the zero calibrator
		(2) The accuracy should be ± 20% of nominal concentration (from ≥ five replicates in at least three runs)
		(3) The precision should be ± 20% CV (from ≥ five replicates in at least three runs)
Accuracy and precision (A&P)	(1) A & P should be established with at least three independent A & P runs, four QC levels per run (LLOQ, L, M, H QC), and ≥ five replicates per QC level	(1) The run should meet the calibration curve acceptance criteria and include the LLOQ calibrator
		(2) This run has no QC acceptance criteria
		(3) Accuracy: Within-run and between runs: ± 15% of nominal concentrations; except ± 20% at LLOQ
		(4) Precision: Within-run andbetween runs: • ± 15% CV, except ± 20% CV at LLOQ
Recovery	(1) Extracted samples at L, M, and H QC concentrations versus extracts of blanks spiked with the analyte post extraction (at L, M, and H)	(1) No acceptance criteria defined
Stability	(1) For auto-sampler, bench-top, extract, freeze–thaw, stock solution and long-termstability, perform at least three replicates at L and H QC concentrations	(1) The accuracy (% nominal) at each level should be ± 15%

However, this level of method validation is not necessarily required for other applications outside the regulatory or clinical environments including the quantification of endogenous metabolites where what is being sought may be confirmation of a hypothesis that certain metabolites are directly associated with a disease and are either a direct cause of the disease or are a direct response to the disease. The UK Consortium on Metabolic Phenotyping (MAP/UK) have discussed these requirements and have adapted FDA, EMA and ICH guidelines to propose a less onerous ‘tiered’ approach to evaluate the reliability of a wide range of metabolomics analyses. This ‘fit-for-purpose’ tiered approach comprises four application levels (discovery, screening, qualification and validation) and is discussed in the context of a range of targeted and untargeted metabolomics assays (Sarmad et al., 2023).

If an assay is not validated, or the validation results are not/cannot be reported for all of the characteristics above then the data are probably not of “regulatory quality” but may still be fit for purpose. To show this is the case the authors should clearly state what the assay purpose was, what was done in terms of validation and report on the validation outcomes (the data and results should be documented and made publicly available either on request to the laboratory, in supplementary materials in a paper when the method is first reported or as a validation report on the laboratory’s website which can be accessed by all external researchers).

The linear calibration range and LOQ/LOD defines the range of concentrations where the metabolite should be assayed, responses below the LOQ/LOD should be reported as < LOD/LOQ and not a concentration (even if a concentration above zero is calculated). Concentrations higher than the upper limit of the calibration curve, which is then effectively the upper limit of quantification (ULOQ) should generally not be reported. However, if the linear range of detection is significantly greater than that of the calibration curve it may be justifiable to extrapolate concentrations above the ULOQ (up to 120% of the top calibrant) whilst clearly reporting them as being outside the linear calibration range. However, best practice would be to dilute samples so that the concentration falls into the linear calibration range and perform reanalysis of the diluted sample. All assays should be validated/cross validated for each sample matrix to be included in a study. Thus the recovery, linear range and LOQ/LOD and ULOQ may differ for the same metabolite in serum and plasma even though the same UHPLC-MS assay is applied; however, cross validation between such similar matrices is less onerous than full validation would be.

## The use of quality control samples in untargeted and targeted studies

Quality assurance (QA) and quality control (QC) processes should be applied in any analytical assay described in this manuscript and is hugely important to be able to demonstrate the consistency and overall quality of data reported (Eurachem, 2016). Different types of QC samples can be applied for untargeted (Broadhurst et al., 2018) and targeted (Food and Drug Administration, 2018) assays along with the use of extraction and solvent blank samples. The use of a Standard Reference Material (SRM) (i.e. a Certified Reference Material produced by the National Institute of Standards and Technology (NIST)) for which metabolite concentrations and/or mass fractions may or may not be established can also be applied in both types of assays (Bowden et al., 2017). A SRM can be applied either to report data quality from replicate extractions and analyses in an untargeted study or to report concentrations relative to the SRM where metabolite concentrations are reported for the SRM (e.g. see SRM1950 available from NIST) for semi-targeted and targeted assays.

In targeted assays, QC samples are applied to demonstrate the accuracy and precision of the method during validation and for each analytical batch during biological sample analysis. The QC samples are ideally constructed in the sample matrix with chemical standards spiked in to prepare a QC sample with known metabolite concentrations. Typically, at least three QC samples are prepared at low, medium and high concentrations (with metabolite concentrations close to the LOQ/LOD, in the middle and towards the top (approximately 80%) of the linear calibration curve (Food and Drug Administration, 2018)). Of course, in multiplexed analyses targeting multiple metabolites these concentrations can be different for each metabolite because their LOD/LOQ and linear calibration ranges can be different.

In untargeted assays and because no concentrations are being reported because the targets are not known, QC samples are applied to demonstrate precision (either for the analytical process of data collection and for sample preparation or for the analytical data acquisition process depending on the sample type). QC samples cannot be applied to demonstrate accuracy. Typically, a pooled QC sample is applied and is prepared by mixing aliquots of a subset or all biological samples to generate a pooled sample which represents the qualitative metabolite composition and the average metabolite concentrations across the biological samples. For biofluid samples the pooled QC will also represent the sample matrix but for cellular and tissue samples where a pooled QC is prepared after sample extraction then this will not be the case. The literature demonstrates that these samples are injected after every 5-20th biological sample, depending often on the size of the analytical batch but, independent of the UHPLC assay type (lipidomics, HILIC, aqueous reversed-phase etc.,). The chromatographic peak area and detection rate are used to assess the quality of the data, specifically the stability of the analysis and the precision of the analyte peak areas (Broadhurst et al., 2018).

## How accurate is your reported concentration?

A number of different experimental factors will influence the accuracy of your reported concentration as have been discussed above. A range of these are described in Table 5 and should be considered when assessing data collected in your laboratory or publicly available data. The impact on the biological conclusions derived from such data should also be considered. If an absolute concentration is reported then the researcher should assess the quality of the method and its validation and how this could bias or impact on the accuracy of the reported concentration on derived biological conclusions. For example, in meta-analyses where concentration data are used, if some studies report accurate concentrations and others report semi-accurate concentrations then the meta-analysis is biased/inaccurate and should not be performed. As discussed in Sect. 3.3, if a concentration is reported then it should not be assumed that it is accurate.

**Table 5:** Factors to be considered when assessing the accuracy of reported concentrations

Experimental factor	Notes
Has the method been validated to demonstrate it is fit-for-purpose including to demonstrate accuracy of reported concentration?	An appropriately validated analysis will demonstrate that high accuracy of reported concentrations is achievable in the sample or surrogate matrix to be applied for calibration solution preparation
Has the linear calibration range and limit of detection of the method been experimentally determined for the instrument to be applied?	A high accuracy concentration can not be assumed with no knowledge of the linear calibration range (and limit of detection)
Is the reported concentration in the linear range of the calibration curve?	If the concentration of the metabolite falls outside the linear calibration range but the equation for the linear calibration range is applied for calculating a concentration then the concentration reported will not be of high accuracy
Is the reported concentration close to the limit of detection?	The accuracy of a reported mass concentration will be lower when close to the limit of detection compared to higher concentrations in the linear calibration range
Has a calibration curve been applied to determine concentration?	If a calibration curve has not been constructed with data from the analysis then a highly accurate mass concentration can not be assumed
Has a single calibration point or multiple calibration point calibration curve been applied?	Six point calibration curves will provide higher accuracy of reported mass concentrations when compared to single point calibration
Has a reference material been applied to determine concentration?	This applies a single point calibrant, see experimental factor above
What is the matrix applied for calibration solution preparation?	The calibration curve parameters can be different for the sample matrix compared to a surrogate matrix
If a surrogate matrix is applied then has this been assessed with sample matrix and is a structurally similar internal standard applied?	The linear calibration range and limit of detection may differ when different matrices are applied. A correction factor can be calculated and applied when a surrogate matrix is applied instead of the sample matrix. An appropriately chosen internal standard will correct for differences in matrices

## Conclusions

Here the principles of different types of assays applied in metabolomics studies, the quantification processes applied in these studies, how each quantification process is applied and how the process applied influences the data reported have been discussed. As the major conclusion, even when a concentration is reported we recommend that the scientist question how the data have been collected, how the concentration has been calculated, whether the assay has been validated (and, if so, how) for the specific metabolite and sample matrix and how these may introduce biases or errors in reported concentration data.

## Data Availability

No datasets were generated or analysed during the current study.

## Declarations

### Research involving human and animal participants

No research involving Human Participants and/or Animals was performed in the writing of this manuscript.
